# Comparison of organ and effective dose estimations from different Monte Carlo simulation‐based software methods in infant CT and comparison with direct phantom measurements

**DOI:** 10.1002/acm2.13625

**Published:** 2022-05-06

**Authors:** Michael Lawson, Kemal Berk, Mohamed Badawy, Yujin Qi, Ahilan Kuganesan, Peter Metcalfe

**Affiliations:** ^1^ Monash Health Imaging Monash Health Clayton Victoria Australia; ^2^ Centre for Medical Radiation Physics, School of Physics, Faculty of Engineering and Information Sciences University of Wollongong Wollongong New South Wales Australia; ^3^ Department of Physical Sciences Peter MacCallum Cancer Centre Melbourne Victoria Australia; ^4^ Department of Medical Imaging and Radiation Sciences, School of Primary and Allied Health Care, Faculty of Medicine, Nursing and Health Sciences Monash University Clayton Victoria Australia

**Keywords:** computational dosimetry, computed tomography, effective dose, infants, organ dose, thermoluminescent dosimeters

## Abstract

**Purpose:**

Computational dosimetry software is routinely used to evaluate the organ and effective doses from computed tomography (CT) examinations. Studies have shown a significant variation in dose estimates between software in adult cohorts, and few studies have evaluated software for pediatric dose estimates. This study aims to compare the primary organ and effective doses estimated by four commercially available CT dosimetry software to thermoluminescent dosimeter (TLD) measurements in a 1‐year‐old phantom.

**Methods:**

One hundred fifteen calibrated LiF (Mg, Cu, P)‐TLD 100‐H chips were embedded within an anthropomorphic phantom representing a 1‐year‐old child at positions that matched the approximate location of organs within an infant. The phantom was scanned under three protocols, each with whole‐body coverage. The mean absorbed doses from 25 radiosensitive organs and skeletal tissues were determined from the TLD readings. Effective doses for each of the protocols were subsequently calculated using ICRP 103 formalism. Dose estimates by the four Monte Carlo–based dose calculation systems were determined and compared to the directly measured doses.

**Results:**

Most organ doses determined by computation dosimetry software aligned to phantom measurements within 20%. Additionally, comparisons between effective doses are calculated using computational and direct measurement methods aligned within 20% across the three protocols. Significant variances were found in bone surface dose estimations among dosimetry methods, likely caused by differences in bone tissue modeling.

**Conclusion:**

All four‐dosimetry software evaluated in this study provide adequate primary organ and effective dose estimations. Users should be aware, however, of the possible estimated uncertainty associated with each of the programs.

## 1 INTRODUCTION

Radiation doses to the patient during computed tomography (CT) procedures are among the highest in diagnostic radiology.^1^ In the USA, CT radiation doses account for over half the population effective dose received from medical sources.^2^ Even with this knowledge, the total number of yearly CT examinations grows.^3^, ^4^ Large‐scale studies have indicated the detrimental impact of ionizing radiation on human tissue, with the increased radiosensitivity of pediatric populations compared to adult populations, an area of particular concern.^5^ During a child's development, cells proliferate more rapidly. Thus, they are more vulnerable to the effects of damage caused by radiation. Additionally, a radiation‐induced malignancy may remain latent for up to 40 years in some disease types. A study by Mathews et al. indicated an increase in cancer incidence for individuals who had a CT examination as a child.^6^ Consequently, a medical practitioner must apply stringent criteria for conducting pediatric CT imaging to ensure benefits of the procedure outweigh the radiation risk.

To assess the risks of radiation effects from CT scans, it is important to determine the doses to individual organs in addition to the effective doses. The mean absorbed dose in an organ, measured in milligray (mGy), is defined as the ratio of the amount of energy deposited by ionizing radiation in the organ to the mass of that organ. The mean absorbed dose is a measurable radiation quantity that can correlate with the radiation risk. The effective dose, expressed in millisieverts (mSv), conveys the overall risk of stochastic effects, such as radiation‐induced cancers.^7^ The effective dose is a calculated quantity and considers the individual tissue sensitivities by applying a weighted sum of absorbed doses over all tissues in the body.

Absorbed dose in an organ and tissue from CT exposure is not readily available from the CT scanner and is complex to directly measure. A comprehensive review of the methodology of estimating patient organ dose with CT scans can be found in the AAPM Report 246.^8^ A direct method for measuring CT organ dose is thermoluminescent dosimeters (TLDs) inside a patient‐equivalent phantom. TLDs are ideal due to their physical and dosimetric characteristics, including small size, high sensitivity, dose and energy linearity, and reusability.^9^ In particular, LiF:Mg,Cu,P (TLD‐100H) are known to possess a superior energy and tissue equivalence compared to other thermoluminescent materials.^10^ Several studies have used TLD‐100H chips to perform CT organ dosimetry within adult and pediatric anthropomorphic phantoms.^11^, ^12^, ^13^, ^14^ Existing computational methods for organ dose estimations are predominantly based on Monte Carlo radiation transport simulations. It can provide an accurate and noninvasive means of estimating the organ and effective doses for a given radiological examination. There are many commercial software programs available, such as CT‐Expo,^15^ VirtualDose,^16^ WAZA‐ARI,^17^ and NCICT,^18^ which contain user‐friendly graphical interfaces that allow the user to match the patient and scanner characteristics (e.g., patient size, scanner manufacturer/model, scan range, and technical parameters) to the desired examination.

The various software operate using a similar methodology, whereby organ dose coefficients normalized to volumetric CT dose index (CTDI_vol_) are established for incremental slice positions for each phantom, tube voltage, and scanner model through Monte Carlo photon transport simulations. The culmination of organ dose coefficients from the included slices (selected by the scan range) and user‐entered CTDI_vol_ determines the total organ dose for a given application. However, variance in scanner modeling, computational phantoms, and photon transport algorithms can lead to considerable variation in organ dose coefficients. In particular, variations in the style and size of the computational phantom's characteristics can significantly impact the estimated organ and effective dose. First‐generation computation software used stylized or mathematical phantoms, the organs of which were created from simplistic three‐dimensional volumes.^19^ This simplification of anatomical structures restricts stylized phantoms’ resemblance to human organs and subsequently may diminish software accuracy. Additionally, the widespread uptake of stylized phantoms is limited to a few age groups and body sizes for adults and pediatrics. Second‐generation phantoms, known as voxelized phantoms, were created through the segmentation of transaxial clinical CT or MRI images. Although this process made the phantoms more anthropomorphous, it limited the resolution of anatomical structures in the coronal and sagittal planes. It also made voxelized phantoms inflexible to desired modifications of organ size or location to reflect the average‐sized patient. Hybrid computational phantoms, the third generation of phantoms, utilize existing voxelized phantoms and apply modeling algorithms to organs to allow greater deformation and anatomical realism.^20^ The main features of these software packages are summarized in Table 1.

**TABLE 1:** Phantom generation and age for computational dosimetry software

	**Country of origin**	**Creator**	**Year of release**	**Phantom generation**	**Phantom creator**	**Selectable phantoms (male and female)**
CT Expo	Germany	G. Stamm, H.D. Nagel	2001	Voxelized	National Cancer Research Centre for Environment and Health	“BABY”—8 week old “CHILD”—7 year old “ADULT”
NCICT	USA	National Cancer Institute	2015	Hybrid	National Cancer Institute	Newborn, 1‐year old, 5‐year old, 10‐year old, 15‐year old, adult
VirtualDose	USA	National Institute of Biomedical Imaging and Bioengineering	2015	Hybrid	RPI and UF	Newborn, 1‐year old, 5‐year old, 10‐year old, 15‐year old, adult
WAZA‐ARI	Japan	Oita University of Nursing and Health Sciences and the Japan Atomic Energy Agency	2012 (v1) 2015 (v2)	Hybrid	Adult Phantoms—Japan Atomic Energy Agency Paediatric Phantoms—the University of Florida and the National Cancer Institute	Newborn, 1‐year old, 5‐year old, 10‐year old, 15‐year old, adult

Abbreviations: RPI, Rensselaer Polytechnic Institute; UF, University of Florida.

Recent comparison studies using different software packages have found significant deviation in the calculated organ and effective doses.^21^, ^22^, ^23^ These have been attributed to variation in anatomy between phantoms and variation in scanner‐matching methods between software programs. However, there have been limited studies assessing pediatric phantoms,^12^, ^24^, ^25^ with no studies published evaluating the accuracy of the dosimetry software for skeletal tissue or effective dose estimates in CT examinations involving 1‐year olds. The objective of this study was to compare the different software for CT primary organ dosimetry and effective dose estimation and to assess their accuracy for clinical application in a 1‐year‐old infant. The calculated organ doses were compared with TLD measurements placed in a physical anthropomorphic phantom representing a 1‐year‐old child, across multiple scan parameters and CT scanners. This study is the first to evaluate the different dosimetry software packages and TLD measurements for skeletal tissue and effective doses.

## 2 METHODS

### 2.1 Thermoluminescent dosimeters

One hundred and fifteen high sensitivity lithium fluoride doped with magnesium, copper, and phosphorous (LiF:Mg,Cu,P) 3 × 3 × 0.89 mm^3^ thermoluminescent chips (TLD‐100H, Harshaw Chemical Company, OH, USA) were used for organ dosimetry. Initially, all TLDs underwent two annealing cycles before using a programmable annealing oven (SEM Company). Individual sensitivity correction factors (SFs) for each TLD were obtained for absorbed dose by delivering a single 200‐mGy dose from a 6‐MV Varian linear accelerator beam (Varian Medical Systems, Crawley, United Kingdom). TLDs were centered 100 cm from the source at a depth of 10 cm in a water‐equivalent phantom (RW3 Slab Phantom) to ensure that a uniform dose was delivered. Readout occurred using an automated Harshaw 5500 unit that heated the TLDs to a maximum temperature of nominally 255°C at a heating rate of 10°C/s to optimize the signal‐to‐noise ratio.^26^ The same read‐out process was used throughout the experiment. SFs were determined by dividing the nano‐Coulomb response for each TLD by the batch average. This process was repeated three times to determine an average response per TLD. Eight TLDs were subsequently removed from the batch as either their SFs varied by more than ±10% from the average batch response or their SFs were not reproducible within ±10%. All remaining TLDs had their sensitivities corrected throughout the experiment.

### 2.2 Phantom and organ dosimetry

**FIGURE 1:**
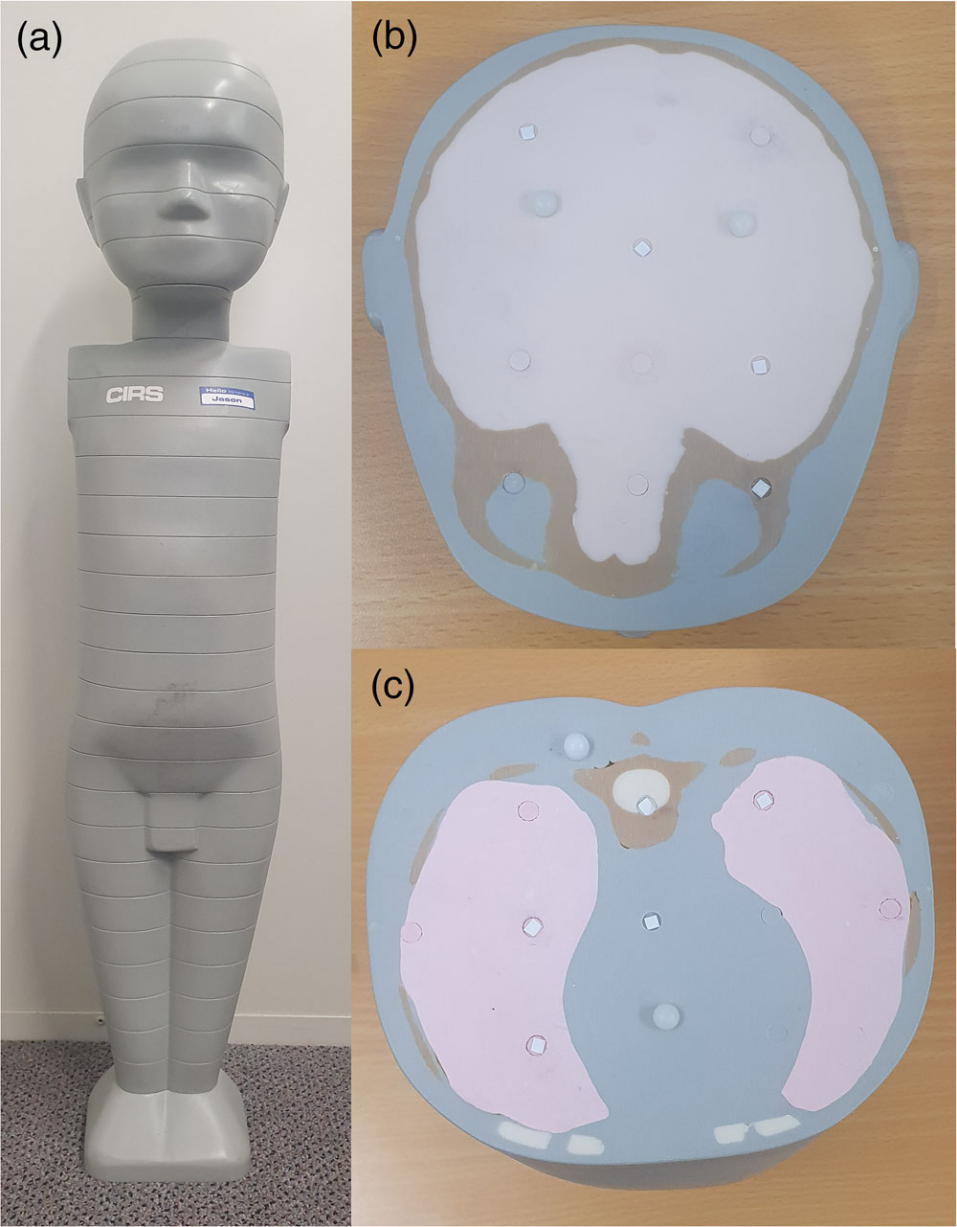
(a) The anthropomorphic phantom used for direct organ dose measurements. (b) The placement of TLDs within the phantom (skull). (c) The placement of TLDs with the phantom (thorax). TLDs, thermoluminescent dosimeters

A CIRS 704 anthropomorphic phantom (CIRS, Inc., Norfolk, Virginia, US) was used to simulate an average 1‐year old. The phantom consists of 25‐mm thick slices with removable inserts for dosimeter placement (Figure 1). A subset of removable inserts were slightly pushed into the adjacent slice to create a small gap for the TLDs to be positioned. Ninety‐seven TLDs were placed throughout the phantom to reflect the anatomical location of different organs (as reflected in Table 2). The TLDs were positioned according to Inkoom's determination of the locations of radiosensitive organs within the CIRS 704 phantom.^27^ The decision on how many TLDs were embedded within each organ was based on the organ's size and the tissue weighting factor.^7^ For calibration purposes, five unexposed TLDs were set aside on each scan. For background radiation determination, another five TLDs were left unexposed.

**TABLE 2:** Placement of TLDs throughout infant anthropomorphic phantom

Anatomical tissue	Number of TLDs
Primary organs	
Brain	7
Lung	12
Stomach	5
Liver	7
Thyroid	2
Salivary glands	4 (2 parotid, 2 sublingual)
Testes	2
Ovaries	2
Bladder	4
Colon	10 (3 ascending, 3, transverse, 2 descending, 2 sigmoid, and 1 rectum)
Active marrow and bone surface	16 (3 legs, 3 pelvis, 2 rib, 4 spine, 4 skull)
Skin	6
Esophagus	3
Breast tissue	2
Remainder organs	
Heart	1
Muscle	Average of limb and torso TLDs
Small intestines	4
Uterus	1
Prostate	1
Kidneys	1
Spleen	1
Additional organs	
Spine	4
Lens	2

Abbreviation: TLDs, thermoluminescent dosimeters.

For each CT scan, the five calibration TLDs were exposed to a known dose from a calibrated Xstrahl orthovoltage source, with an applied tube voltage of 120 kVp and a halve value layer thickness of 5 mm of aluminum. The calibration exposure parameters (kVp and HVL) were chosen to ensure a close match in each protocol of the unattenuated beam characteristics produced by the CT. In each calibration event, 0.5 Gy was delivered to each of the TLDs. An average calibration factor (CF) in gray per coulomb (Gy/C) was subsequently calculated by dividing the nominal dose by the average TLD response. The dose for each organ‐based TLD was computed by multiplying the TLD's response (*r*) by its individual SF and the batch CF and subtracting the background TLD readings (*b*), as shown in the following equation:

(1) $$D_{n}=SF_{n}\left(r_{n}-b\right)\times CF\;$$

### 2.3 Scan protocols

To assess the doses to each organ at risk from primary beam irradiation, the CIRS phantom underwent a vertex to toe single helical acquisition. Multiple scanners and tube voltages were used to comprehensively examine the doses under different conditions. A Canon Aquilion One Vision (Canon Medical Systems Corporation, Otawara, Tochigi, Japan) and GE Discovery 750 HD (GE Healthcare, Chicago, Illinois, United States) each had acquisition protocols selected. The tube voltages, collimations, and helical pitches selected for each protocol were chosen to reflect the local site's parameters for clinical infant torso CT examinations on each scanner. An additional acquisition at 100 kVp was also performed on the GE Discovery scanner (protocol 2). Table 3 displays the scanner parameters used for each acquisition.

**TABLE 3:** CT acquisition scan parameters

Protocol	Manufacturer/make	Tube voltage (kVp)	Tube current (mA)	Tube rotation (s)	Pitch	Collimation (mm)	CTDI_vol_ (mGy)
1	GE Discovery	80	800	0.4	0.97	32 × 0.625	8.89
2	GE Discovery	100	800	0.4	0.97	32 × 0.625	16.65
3	Canon Aquilion One Vision	80	700	0.275	0.83	80 × 0.5	7.50

Abbreviations: CT, computed tomography; CTDIvol, volumetric computed tomography dose index.

To ensure that the only contribution to the organ doses was from the helical acquisition, TLDs were not embedded within the phantom during the planning (scout) scans. Most computational software packages do not have the functionality to estimate the dose associated with planning scans and hence were not considered as part of this study. The TLDs were instead carefully inserted after alignment and planning scans had been completed. The planning scans were used to prepare the scan coverage for the main acquisition. The filter selected was based on the smallest scan field of view, which encapsulates the entire phantom, as would be done clinically. Additionally, the tube current for each scan was significantly increased (without modulation) from the clinical protocol to increase the dose to the TLDs and reduce measurement uncertainty. Each scan was repeated twice, with the TLDs being read out after each acquisition to ensure the precision and accuracy of the dose measurements. Any TLD readings that varied by more than 20% between the two sets of scans were repeated a third time to remove outlying measurements from the dataset.

### 2.4 Organ and effective doses

#### 2.4.1 Tissue absorption correction

As the TLDs were calibrated in dose to water, a factor was required to correct for the difference in radiation absorption among different tissues in the body. As the range of photoelectrons generated at diagnostic energies is much smaller than the minimum dimensions of the TLDs, the Bragg–Gray cavity theory cannot be assumed as photoelectrons generated by photon interactions within the TLD itself contribute to most of the dose. Therefore, a ratio of the mass attenuation coefficients is considered more appropriate. The dose to a given tissue can be calculated by the following equation:

(2) $$D_{T}=\bar{D_{W}}\;\times \;\frac{{\left[\frac{\mu_{en}\left(E\right)}{\rho }\right]}_{T}}{{\left[\frac{\mu_{en}\left(E\right)}{\rho }\right]}_{W}}$$

where $\left[\frac{\mu_{en}\left(E\right)}{\rho }\right]$ is the mass absorption coefficient at a given energy (*E*) for a given tissue as defined by ICRU 46,^28^ $D_{W}$ is the average measured dose to water, and $D_{T}$ is dose converted to the specific tissue type.

As a CT beam consisted of a polychromatic spectrum, the weighted correction was applied over the range of spectral energies that accounted for the different photon energies prevalence within the CT beam. SpekCalc Software v1.1 (The Institute of Cancer Research London, United Kingdom) simulated each protocol's photon energy spectrum, by inputting the nominal tube voltages and known filter characteristics.^29^ The effective energy of each of the unattenuated CT spectrums matched the calibration spectrum with ±5 keV. No adjustment was made for phantom scatter and spectral hardening due to phantom attenuation.

The mean organ doses were calculated using both sets of TLD measurements for each protocol. The uncertainties in a protocol's organ doses were calculated as the first standard deviation of all dose measurements within the given organ from both sets of TLDs. Tissue doses were determined by calculating the average tissue corrected dose across each TLD within the organ, except for the colon, muscle, skin, active bone marrow, and bone surface that required additional considerations. The total dose to the colon was calculated by the sum of each section's dose, weighted by its contribution to the overall mass of the colon as defined by ICRP 23.^30^ For simplicity, the calculated dose to the muscle tissue was approximated as the average dose across all TLDs within the torso and legs. The average dose to the skin was calculated using six TLDs wrapped in a thin layer of low‐density polyethylene and evenly distributed TLDs on the surface of the phantom.

#### 2.4.2 Skeletal dosimetry

Due to the intricate and macroscopic structures surrounding bone marrow, the dosimetry associated with the tissue remains complex. Two types of bone marrow exist in most age groups: red and yellow. All bone marrow that an individual is born with is red; however, the marrow turns from red to yellow as the individual ages. In an infant of age 1 year, most of the total marrow is still considered red marrow. Red bone marrow (RBM) is hemopoietically active and is considered highly radiosensitive, whereas yellow bone (inactive) marrow is occupied by mostly fat cells and is considerably less radiosensitive. Active bone marrow occupies the cavities within long bones and fills the space left by trabecular bone in the cranium, pelvis spine, and vertebra. Photoelectrons that originate from the more highly attenuating trabecular bone and that deposit dose through secondary electron interactions in adjacent marrow tissue have a significant impact on the total dose deposited in RBM and, therefore, need to be considered.^31^ King et al. photon energy and bone site‐specific RBM dose enhancement factors (DEFs) for a 1.7‐year old were applied in this study to account for this additional photoelectron contribution to RBM.^32^

The shallow marrow is regarded as the surrogate tissue for the osteoprogenitor cells. The ICRP 110 publication defines shallow marrow as the total marrow (both active and inactive) within 50‐μm distance from the bone surfaces.^33^ As the phantom is not equipped with TLD positions at the locations analogous to the shallow marrow of bones, the TLD raw dose readings used for active marrow dosimetry were also used for shallow marrow dosimetry. A similar but more pronounced shallow marrow‐specific DEFs (due to the thickness and closer proximity of the total marrow to the trabeculae surfaces) were applied to account for the increased photoelectron dose contribution to the shallow marrow. Because comprehensive pediatric shallow marrow DEFs are yet to be published, adult shallow marrow DEFs published in ICRP 110 were used in this study.^33^

The RBM and shallow marrow doses at each skeletal site were calculated individually. The total RBM and shallow marrow doses were calculated by a weighted sum, based on the skeletal site's RBM or shallow marrow mass relative to the total RBM or shallow marrow mass throughout the entire skeleton. Shallow marrow mass absorption coefficients also accounted for the cellularity (ratio of RBM to total marrow) at each skeletal site.^34^ The following equation was used to determine the phantom's RBM and shallow marrow doses^44^:

(3) $$\begin{array}{ccc} D_{RBM/SM} & = & \sum_{i}^{n}\left[\bar{D_{W,\;i}}\times \;\frac{{\left[\frac{\mu_{en}}{\rho }\right]}_{RBM/SH}}{{\left[\frac{\mu_{en}}{\rho }\right]}_{W}}\right.\\ & & \left.\times \left(1+S{\left(E\right)}_{i}\right)\times \;\frac{M_{i}}{M_{total}}\right]\; \end{array}$$

where *S*(*E*)*_i_* is the RBM or shallow marrow DEFs for the skeletal site *i*, *M_i_* is the mass of RBM or shallow marrow or in the given bone, and *M_total_* is the total mass of RBM or shallow marrow in a 1‐year old as defined in the ICRP publication 70 and 89.^34^, ^35^, ^36^ In this study, shallow marrow shall be known as “bone surface” in‐line with the ICRP 103 report formalism.^7^

Effective doses were calculated using the ICRP 103 methodology and published tissue weighting factors for each organ dose.^7^ As not all “remainder organs” could be measured, the dose was assumed to be an average of all measured remainder organ tissues. The effective dose was calculated individually for each sex using the testes and prostate doses for male calculations, and ovaries and uterus doses for females. The standard errors associated with effective dose estimates were calculated according to Bevington et al.^37^ Organ and effective doses for each protocol were normalized to CTDI_vol_ and presented.

### 2.5 Software dosimetry

Individual dose simulations were performed by CT‐Expo, NCICT, VirtualDose, and WAZA‐ARI dosimetry software applying the same scan parameters used in the phantom measurements. This included the CT model, tube voltage, pitch, collimation, and CTDI_vol_. Each of the software had a range of computational phantom choices, and the most applicable one to 1‐year‐old dosimetry was selected for each software. The similarities between computational phantoms are indicated in Table 4. For NCICT, VirtualDose, and WAZA‐ARI, this corresponded to a “1‐year old” phantom selection. For CT Expo, no available computational phantoms directly correlated to a 1‐year‐old equivalent phantom; instead, the “BABY” phantom was selected as the closest available alternative, representing an 8‐week‐old infant. Doses were also calculated for both male and female phantoms. Aside from gonadal and reproductive organs, the individual organ doses varied by less than 5% between male and female phantoms and were, therefore, averaged to provide a hermaphroditic organ dose that could be easily compared to TLD measurements of the hermaphrodite phantom. Prostate, testes, ovary, and uterine organ doses were not included in this process. For CT‐Expo, breast tissue dose was not averaged across sexes, as male breast doses were calculated as 0 mGy. Additionally, computationally determined effective doses for each sex were directly compared to TLD‐measured effective doses. Percentage variation in organ and effective doses between software methods (*D_soft_*) and direct TLD measurements (*D_TLD_*) were calculated by the following equation^38^:

(4) $$Variation\;\left(\%\right)=\frac{D_{Soft}-D_{TLD}}{D_{TLD}}\;\times 100$$

**TABLE 4:** Size characteristics for physical and computational phantoms compared to a 1‐year‐old child

Characteristic	Average dimensions of a 1‐year‐old child^38^	Physical phantom (CIRS 704)	CT Expo	NCICT	Virtual‐dose	WAZA‐ARI
Weight (kg)	Male: 9.6 Female: 9.0	10	4.2	10	9.39	10
Height (cm)	Male: 76 Female: 74	75	57	76	76.6	76
Age	–	1‐year old	8‐week old	1‐year old	1‐year old	1‐year old

## 3 RESULTS

Tables 5 and 6 show the organ and effective doses from TLD measurements normalized to CTDI_vol_ (32‐cm reference phantom), respectively. Doses were normalized by CTDI_vol_ as it accounts for not only tube current time, but also variations in pitch. There was a close agreement between organ and effective doses per CTDI_vol_ between protocols. Small variations in organ doses may be attributed to differences in spectral output caused by different X‐ray tube characteristics and filtrations.

**TABLE 5:** TLD‐derived (mean ± 1 SD) primary organ doses per CTDI_vol_ (mGy/mGy) for each whole‐body CT protocol

Organ	CTDI_vol_ normalized organ dose (mGy/mGy)		
	Protocol 1	Protocol 2	Protocol 3
Active marrow	1.67 ± 0.29	1.81 ± 0.29	1.98 ± 0.46
Bladder	2.16 ± 0.25	2.03 ± 0.18	2.4 ± 0.29
Bone surface	1.67 ± 0.51	2.78 ± 0.31	2.72 ± 0.38
Brain	1.71 ± 0.12	1.86 ± 0.18	2.16 ± 0.17
Breast	2.01 ± 0.16	1.82 ± 0.09	2.14 ± 0.10
Colon	2.2 ± 0.08	1.98 ± 0.22	2.19 ± 0.23
Liver	2.11 ± 0.34	2.26 ± 0.12	2.38 ± 0.14
Lung	2.43 ± 0.3	2.46 ± 0.25	2.52 ± 0.39
Esophagus	2.28 ± 0.31	2.19 ± 0.17	2.46 ± 0.02
Ovaries	1.94 ± 0.22	1.99 ± 0.19	2.21 ± 0.24
Salivary glands	1.8 ± 0.12	2.09 ± 0.21	2.39 ± 0.07
Skin	2.48 ± 0.24	2.37 ± 0.43	2.79 ± 0.42
Stomach	2.06 ± 0.2	2.2 ± 0.18	2.28 ± 0.23
Testes	2.55 ± 0.22	2.34 ± 0.37	2.43 ± 0.13
Thyroid	2.69 ± 0.23	2.56 ± 0.54	3.4 ± 0.16
Heart	2.36 ± 0.14	2.33 ± 0.24	2.65 ± 0.06
Kidney	2.36 ± 0.22	2.11 ± 0.28	2.16 ± 0.08
Muscle	2.12 ± 0.28	2.1 ± 0.18	2.38 ± 0.42
Pancreas	2.35 ± 0.17	2.19 ± 0.22	2.37 ± 0.11
Prostate	2.1 ± 0.12	1.98 ± 0.06	2.27 ± 0.2
Small bowel	1.94 ± 0.20	1.97 ± 0.27	2 ± 0.17
Spleen	2.48 ± 0.26	2.1 ± 0.17	2.74 ± 0.12
Uterus	1.79 ± 0.11	1.98 ± 0.06	1.94 ± 0.16
Lens	1.78 ± 0.13	1.79 ± 0.22	2.25 ± 0.41
Spine	1.92 ± 0.28	1.82 ± 0.21	1.83 ± 0.66

Abbreviations: CT, computed tomography; CTDIvol, volumetric computed tomography dose index, TLD, thermoluminescent dosimeter.

**TABLE 6:** TLD‐derived (mean ± 1 SD) effective doses per CTDI_vol_ (mSv/mGy) for each whole‐body CT protocol

Organ	CTDI_vol_ normalized effective dose (mSv/mGy)		
	Protocol 1	Protocol 2	Protocol 3
Effective dose—male (mSv)	2.15 ± 0.08	2.14 ± 0.05	2.34 ± 0.06
Effective dose—female (mSv)	2.09 ± 0.08	2.12 ± 0.03	2.31 ± 0.09

Abbreviation: CT, computed tomography, CTDIvol, volumetric computed tomography dose index, TLD, thermoluminescent dosimeter.

Despite the tube output remaining consistent throughout each scan, the thyroid, testes, skin, and bone surface consistently received the highest organ doses. The skin's, testes’, and thyroid's proximity to the surface likely attributed to this outcome. The high bone surface dose is likely caused by an elevated DEF at low photon energies, resulting in a greater dose being deposited.

### 3.1 Variations in organ and effective doses between dosimetry methods

**FIGURE 2:**
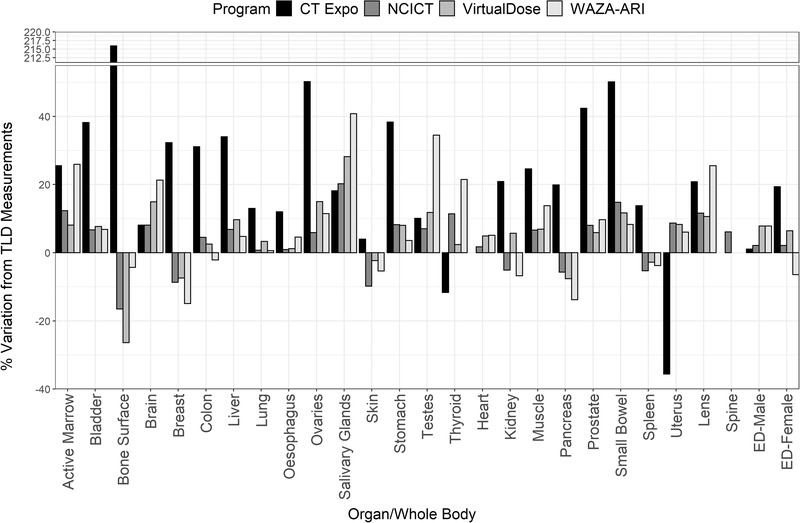
Variation in organ and EDs between dosimetry software and TLD measurements for protocol 1 (80‐kVp GE Discovery). EDs, effective doses; TLD, thermoluminescent dosimeter

**FIGURE 3:**
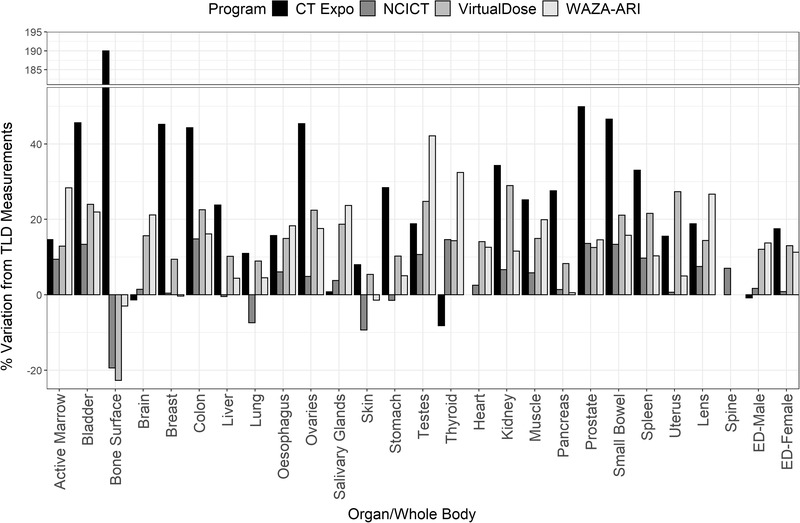
Variation in organ and EDs between dosimetry software and TLD measurements for protocol 2 (100‐kVp GE Discovery). EDs, effective doses; TLD, thermoluminescent dosimeter

**FIGURE 4:**
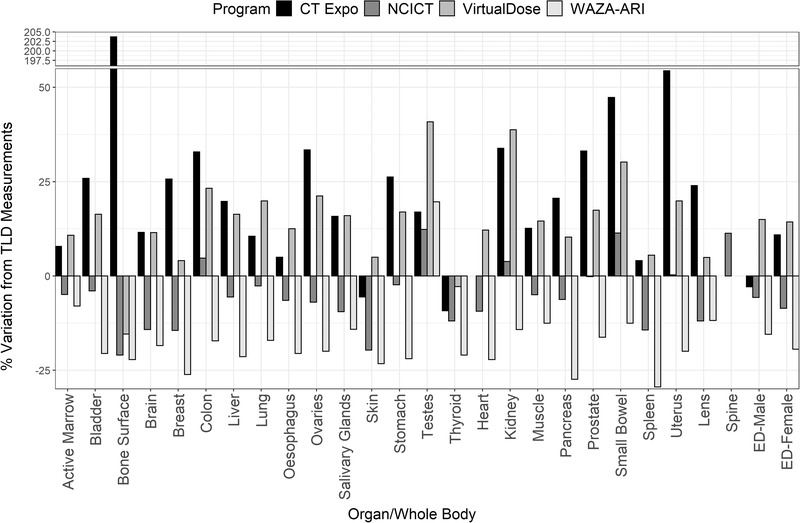
Variation in organ and EDs between dosimetry software and TLD measurements for protocol 3 (80‐kVp Canon Aquilion One). EDs, effective doses; TLD, thermoluminescent dosimeter

Figures 2, 3, 4 show the variation in primary organ doses determined by each dosimetry software compared to the organ dose as measured in phantom for each protocol. Overall, there was a close agreement between TLD measurements and computationally derived doses for most organs and software. For protocols 1 and 2 (Figures 1 and 2), most organ doses estimated by the software were slightly higher than those determined by the physical measurements. For protocol 3, most organ dose measurements from NCICT and WIN‐AZARI were marginally less than TLD measurements, whereas CT‐Expo and VirtualDose were slightly greater than TLD measurements. Bone surface proved to have the most significant variation in estimated dose among the methods, with CT‐Expo, NCICT, VirtualDose, and WAZA‐ARI having a 215%, 16%, 26%, and 5% variation from TLD measurements, respectively. The brain and organs in the thorax, such as the lungs and heart, showed the highest agreement between dosimetry methods. Additionally, NCICT was the only dosimetry software that calculated the dose to the spinal cord, and CT Expo did not calculate the dose to the heart. The average variation to TLD measurements across all organs and protocols was 32%, 8%, 14%, and 16% for CT‐Expo, NCICT, VirtualDose, and WIN‐AZARI, respectively. However, the average variation in CT‐Expo's measurements was reduced to 20% when excluding the bone surface comparison from this calculation.

Figures 2, 3, 4 also show the estimated effective dose given by each dosimetry method for female and male patients. Overall, there was a high level of agreement between effective doses calculated using commercial dosimetry software and TLDs with a maximum variation of 20% and 15% for males and females, respectively. Protocol 1 had the least variation in dose estimates for both males and females, whereas protocol 3 had the greatest variation. As expected, protocol 2 delivered the highest patient effective dose due to the higher CTDI output from a greater tube voltage. All calculation methods except for CT‐Expo indicated a higher effective dose to males when compared to females for equal scan protocols. However, CT‐Expo's male effective dose estimates for all protocols provided the greatest agreement with TLD results (average variation of 1.6% across the three protocols). NCICT's female estimates for all protocols provided the greatest agreement with TLD results (average variation of 3.5%) and aligned well with male estimates (average variation 3.2%). Both VirtualDose and WAZA‐ARI provided dose estimates that aligned closely with TLD results for protocol 1 but showed greater variation for protocols 2 and 3 for both male and female estimates.

## 4 DISCUSSION

This study aimed to compare organ and effective doses estimated using different dosimetry software to TLD measurements performed with a 1‐year‐old phantom for a range of scan protocols and CT scanners. To the best of the author's knowledge, this study is the first to evaluate the different dosimetry software packages and TLD measurements for skeletal tissue doses and effective doses in 1‐year olds.

### 4.1 Findings

Organ doses estimated by NCICT aligned best with TLD results, with an average variation of 8%. Conversely, organ doses estimated by CT‐Expo varied significantly from TLD measurements, particularly for bone surface and torso organs (average deviation of 32%). The variation in bone surface dose can be attributed to CT‐Expo using a different surrogate tissue for the osteoprogenitor cell dose calculation. NCICT, VirtualDose, WIN‐AZARI, and TLD measurements use inactive and active marrow located within 50 μm from the bone surfaces as the surrogate tissue. CT‐Expo assumes a tissue of hard cortical bone, which holds much higher attenuation properties and significantly overestimates the dose to the osteoprogenitor cells.

CT‐Expo's overestimation of absorbed doses to torso organs, such as the colon, liver, and small bowel, is likely due to the inability to select a computational phantom that accurately represents the physical stature of a 1‐year‐old infant. The voxelized MIRD “BABY” phantom was designed to mimic the physical dimension of an 8‐week‐old newborn. Smaller bodies will inherently absorb a proportionately higher radiation dose for a given CTDI_vol_ due to a smaller target mass and laws of exponential attenuation. Therefore, simulations performed on smaller phantoms are likely to overestimate the dose absorbed to torso organs. However, due to an infant's head dimensions changing less rapidly during childhood, organs located within the head are less impacted by imperfect phantom selection. Across the three protocols, CT‐Expo's dose estimate of the brain varied by an average of 7%, significantly less variation than what was recorded for organs within the torso.

VirtualDose and WAZA‐ARI provide estimates that closely align with TLD measurements, with the variation of most organ doses being less than 20%. VirtualDose's dose estimates of the heart, breast, and pancreas aligned within 10% across all protocols. The WAZA‐ARI's calculation of bone surface dose was the computational dosimetry method that matched most closely the TLD‐derived results.

Phantom measurements and all dosimetry software except CT‐Expo indicated a higher overall effective dose for males than for females. This finding is likely due to male gonads receiving a higher absorbed dose than female gonads because of the testes’ proximity to the skin surface. CT‐Expo's contradictory estimate is expected due to the association of zero organ dose to breast tissue for males, significantly decreasing the overall effective dose due to the high tissue weighting factor associated with breast tissue. Interestingly, however, CT‐Expo showed the greatest agreement to TLD measurements for the effective dose calculations in males. However, this was likely caused by the balance of over‐ and underestimation of individual organ‐absorbed doses.

Overall, the findings of this study suggest that organ doses estimated with software that employ 1‐year‐old pediatric phantoms (NCICT, VirtualDose, WAZA‐ARI) provide the greatest agreement with TLD results for infant dosimetry. These hybrid phantoms also bear the greatest resemblance to realistic anatomical features, allowing the user to better individualize the dosimetry based on the patient's physical characteristics. Furthermore, these software packages use the new formalism for bone surface, whereas dosimetry software packages that use the MIRD “BABY” phantom (CT‐Expo) assume that the bone surface tissue is composed of cortical bone, which decreases the accuracy associated with these estimates. However, all four‐dosimetry software packages evaluated in this study provide adequate primary organ and effective dose estimations for the purposes of conveying the stochastic risk for an average reference patient. Due to known limitations with the definition of effective dose, dosimetry software should not be used to calculate an individual's stochastic risk.^39^

### 4.2 Implications

The radiation doses associated with CT procedures and their yearly volume make CT one of the most significant contributors of ionizing radiation to the general population. This is particularly pertinent in highly radiosensitive infants as large‐scale studies indicate an increased stochastic risk after CT examinations.^6^ Therefore, the radiation dose associated with CT examinations should be continuously optimized to minimize stochastic risk during diagnostic CT procedures. To facilitate dose optimization, clinicians must have access to accurate and reliable dose estimation tools. Quantifiable dose optimization requires an efficient means of accurately estimating patient organ and effective doses for given CT examinations.

This study indicates that all dosimetry software packages analyzed provide a reasonably accurate organ and effective dose estimate across the range of scan protocols and CT manufacturers assessed. Regardless of the dosimetry software employed for the dose estimations of infants CT examinations, users should be aware of the variation in dose estimates between software. Effective dose estimates conducted by different dosimetry software have noted differences of 25% between software and as high as 55% and 210% for soft tissue organ and skeletal tissue dose estimates. This finding is consistent with studies looking at variation in effective dose estimates in the adult population.^21^, ^40^ However, only a single published study has compared a subset of TLD infant organ doses to computational dosimetry software and only for a single acquisition protocol. When normalizing organ doses to CTDI_vol_, most organ doses align with measurement uncertainty between the studies. Doses to skeletal tissues and effective doses were not evaluated in that study.^24^

Although whole‐body CT acquisitions in infants are currently limited to a few applications,^41^, ^42^, ^43^ the CTDI_vol_ normalized data presented in this study can easily be implemented to assess the doses to primary beam organs most at risk during infant examinations that implement different scan coverages. This may be particularly useful for clinicians who may not have access to CT dosimetry software. Future studies should consider assessing the accuracy of organ doses outside the primary scan range.

### 4.3 Limitations

This research has several limitations that should be considered in further research. First, the physical phantom constraints limit a definitive judgment regarding the supremacy of a single computational software. The phantom used in this study did not include arms that may have a radiation‐shielding effect on the organs located within the torso. Additionally, all organs and tissues (except for the brain, lung, and skeleton) throughout the phantom were created with a homogeneous soft tissue–equivalent density. Denser torso tissues such as the liver would typically have a slight shielding effect on surrounding tissues such as the stomach that cannot be accounted for in this study. The homogeneous density throughout the torso also meant that hollow structures such as the stomach wall and colon were regarded as solid organs. Second, the dosimetry methodology also had relevant limitations. Although high sensitivity 100‐H (LiF:Mg,Cu,P) TLDs have a smaller energy dependence in the diagnostic energy range than other types of TLDs, there is still an uncertainty caused by the difference in spectral output between the CT protocols and the orthovoltage calibration source. Also, the lack of published DEFs for the shallow marrow dosimetry of pediatrics remains an ongoing issue for all pediatric skeletal dosimetry calculations. Although the assumption that adult DEFs apply for infants may have an associated inaccuracy, other methods that assume a homogenous bone composition and densities are likely to cause even greater uncertainty.^44^

## 5 CONCLUSION

Computational dosimetry software continues to be the preferred method for determining CT doses in clinical settings due to time efficiency and an easy‐to‐use interface. All four computational dosimetry software evaluated in this study provided organ and effective dose estimates, which aligned well to direct TLD phantom measurements across the three clinical CT protocols. Bone surface dose estimations were shown to have the largest variation among methods, likely caused by differences in the target tissue. Although dosimetry software that employs hybrid computational phantoms had a greater congruence with TLD measurements, all software evaluated in this study provided a sufficiently accurate estimate for stochastic risk evaluations in infant CT examinations.

## CONFLICT OF INTEREST

No conflicts of interest to disclose.

## AUTHOR CONTRIBUTION

All authors have contributed to the manuscript—including the development of hypotheses, design, and drafting of the paper. The named authors have no conflict of interest financial or otherwise to disclose.
